# Financing climate justice in the European Union and China: common mechanisms, different perspectives

**DOI:** 10.1007/s10308-021-00644-0

**Published:** 2021-12-04

**Authors:** Stephen Minas

**Affiliations:** 1 School of Transnational Law, Peking University, Shenzhen, China; 2 Transnational Law Institute, Dickson Poon School of Law, King’s College London, London, UK

## Abstract

Climate justice is a concept with many different and competing interpretations. It has salience at intra-country, inter-country and intergenerational levels of climate politics. While inter-country climate justice has long been on the agenda of United Nations climate negotiations, the intra-country and intergenerational aspects of climate justice have assumed new prominence in many countries in recent years, as the economic consequences of mitigation became felt and transnational activism highlighted youth concerns. The diverse elements of and approaches to climate justice have this in common: realising them requires massive financial interventions and reforms. This article examines the still emerging frameworks to finance climate justice in two of the jurisdictions most important to the global response to climate change: the European Union and the People’s Republic of China. The EU and China have in common that they are both on the front line of financial innovation to respond to climate change. They are utilising similar tools of systemic financial intervention in order to transition financing to climate-friendly investment, in the first case domestically, but with clear implications for global financial markets. However, the EU and China are utilising climate financing mechanisms in the context of very different prevailing perspectives on climate justice. This article interrogates the relationship between these different perspectives on climate justice and the distribution, scale and pace of climate finance. The article also observes that while the EU incorporated climate justice considerations in its economic responses to the COVID-19 pandemic with a recovery package prioritising climate action, China did not take the opportunity to foster a ‘green recovery’.

## Introduction

China and the European Union have both extensively reformed their climate policy and legal frameworks in recent years—work that is ongoing in both cases. In both economies, policy frameworks respond to and incorporate notions of ‘climate justice’. As will be seen, the EU and China are developing climate policy under the influence of differing perspectives on climate justice. Nevertheless, in the area of finance, they are employing some similar tools to achieve their stated goals. This article focuses on the relationship between approaches to climate justice in policy frameworks of the EU and China and the emerging frameworks of ‘sustainable’ or ‘green’ finance on both sides.

Sustainable finance may often appear to be a topic of specialised interest for financial officials, regulators and market actors, but it is in fact inseparable from the moral imperative of climate justice. This connection is made directly in the Paris Agreement long-term goal of ‘[m]aking finance flows consistent with a pathway towards low greenhouse gas emissions and climate-resilient development’ (Article 2.1(c)). This goal requires nothing less than that the ‘ultimate purpose of the global financial system’ (which the system is still failing to meet) becomes ‘to ensure the cost-efficient and effective channeling of global savings to finance tomorrow’s inclusive, sustainable economy’ (Zadek 2019, 19–20). This would amount to a normative reorientation of the financial system away from traditionally dominant (at least in the West) accounts of its purpose as a neutral and efficient allocator of resources (Andreotti 2017, 25–27). Article 2.1c has been rightly described as a goal of ‘transformative potential’ (Zamarioli et al 2021, 578), which is different in kind from quantitative pledges of climate finance which, however significant to their recipients, nevertheless amount to a ‘small proportion’ of overall global finance flows (SCF 2018, 12–13). It is no coincidence that ‘mission-oriented’ approaches to finance (Mazzucato 2021), such as the Article 2.1(c) goal, gained ground following the 2007–2008 financial crisis and its exposure of ‘blind faith’ in efficient markets (Jordan 2014, 7).

Continuing failure to make global finance flows consistent with a climate-safe future would perpetuate the injustice suffered by those least responsible for climate change and least able to adapt to it. This means that while most climate financing facilities are not explicitly identified with questions of justice (there are exceptions, such as the EU’s Just Transition Mechanism), the scope and impact of financial interventions are key determinants of climate justice outcomes. In this context, the sustainable finance frameworks of the EU and China are worthy of study not just because of their importance to the achievement of climate objectives in both economies, but also for their capacity to influence other countries and transnational markets.

The remainder of the article is structured as follows. The “Climate justice: multiple levels, contested narratives” section discusses the concept of climate justice and its multiple levels (intra-country, inter-country and intergenerational) and diverse narratives. The “European Union” section begins by examining the incorporation of climate justice concerns in EU climate policies and then demonstrates how financial policies and mechanisms are being employed to implement these policies. The “China” section conducts the same exercise for China, contrasting its ‘ecological civilisation’ policy with the EU’s climate justice policies and also the development of its green finance frameworks. The “Comparing the EU and Chinese financial frameworks” section briefly takes stock of the main similarities and differences of the EU and Chinese financial frameworks. The “Climate justice at the COVID crossroads” section discusses the diverging reactions of the EU and China to the economic and social shock of the COVID-19 pandemic, with major implications for climate policy. The “Conclusion” section concludes that while China and the EU may be utilising a similar set of measures to make their financial systems more sustainable, these measures respond to different prevailing approaches to climate justice in the two jurisdictions. Overall, the EU approach is more closely aligned with the Paris Agreement goal of transforming finance flows, particularly following the announcement of COVID-19 economic packages in both jurisdictions.

## Climate justice: multiple levels, contested narratives

The concept of climate justice invites focus on the injustice suffered by people who are most affected by climate change and least responsible for its causes. This approach can be seen, for example, in the declaration of the 2004 Conference on Environmental Justice and Climate Change (Climate Justice Declaration 2004). However, while climate justice’s meaning may appear self-evident when stated in these terms, both academic literature and political debate reveal it to be contested. Different approaches are informed by competing political and economic schools of thought, as well as different communities within which climate justice is sought (e.g. national communities, the international community, humanity including future generations and nature in general).

Distinctions can be drawn between liberal and eco-centric visions of climate justice. The liberal tradition includes Mill’s harm principle, emphasising a duty to contemporaries, and Rawls’ ‘just savings principle’, which conceptualises a duty to future generations. Liberal political philosophy focuses on distributional choices within and among human communities. Eco-centric approaches stress the need to achieve justice also for non-human animals or for the entirety of nature and seek to widen the ‘speech community’ for this purpose (Eckersley 2004, 123ff). Eco-centric approaches may be discerned in recent constitutional reforms, legislation and court judgements that declare the legal personality or rights of aspects of nature (O’Donnell and Talbot-Jones 2018). It will be seen that both EU and Chinese policies are essentially anthropocentric (although elements of ecocentrism can be discerned in the discussion of ‘ecological civilisation’ within China.) While this article briefly considers the principles informing EU and Chinese approaches to climate justice, it is essentially focused on the ‘procedural dimension of *how* [climate justice] can be achieved politically’ (von Lucke, et al. 2021: 16, emphasis original), i.e., how financial policies and laws in both jurisdictions target outcomes which are consistent with climate justice.

Further distinctions have been drawn regarding the kinds of responsibilities that climate justice entails. Caney (2014, 125ff) has divided these between the responsibilities of ‘burden-sharing justice’ (each state contributing fairly to mitigation) and ‘harm avoidance justice’ (each state doing what they can, beyond domestic mitigation, to prevent catastrophe). Scott has summarised this distinction as ‘do your share and encourage/induce others to do theirs to protect the potential victims of climate change’ (2015, 98). The ‘second-order responsibilities’ of avoiding harm are discharged on the international plain, through activities such as enforcement, incentivisation, enablement, norm creation and undermining resistance to action (Caney 2014, 136–39). It will be seen that the EU is actively addressing both kinds of climate justice responsibility, while China remains more focused on the first. While the EU has sought to accelerate and widen its domestic climate transition, it has simultaneously worked to influence climate outcomes beyond its borders through a growing range of bilateral, inter-regional and global tracks. In contrast, China has been overwhelmingly focused on domestic climate policy, an approach which has produced the contradiction of increasingly ambitious domestic targets set against the high carbon intensity of many Belt and Road projects (Minas 2020). Xi Jinping’s September 2021 announcement that China will no longer build coal-fired power projects overseas is a major development in this regard.

There are also distinctions between the different levels on which climate justice is debated. The intra-country debates over climate justice concern the allocation of costs and support related to the climate transition within polities (FEPS 2019, 7ff). The just transition concept has become prominent in climate justice debates in many countries. These debates are also played out at the EU level among Member States.

The intergenerational dimension of climate justice has long been acknowledged in theory and in rhetoric but has arguably become politically salient only in the last few years. The transnational movement of climate ‘school strikes’ has clearly had an impact on policy, at least in the EU. European Commission Executive Vice-President for the European Green Deal (EGD) Frans Timmermans has stated that ‘[t]he fact that we put sustainability and the climate crisis front and centre [of the Social Democrats’ 2019 European Parliament campaign] is to a large extent the result of our interaction with the young people then and the discussions we had with young people’ (FEPS 2020, 65).

Recent writing on climate justice has emphasised the need to address inter-country, intra-country and intergenerational levels simultaneously. Such an approach is deemed necessary given the sometimes competing claims of different levels of climate justice (von Lucke 2019: 4). The European Economic and Social Committee’s (EESC) 2017 Opinion on Climate Justice highlighted the ‘need to consider the fairness of the often disproportionate impact of climate change on citizens and communities in both developing and developed economies’ (European Economic and Social Committee 2017, 3.2). A Liberal International declaration issued in the context of the 2019 New York climate summit, the ‘Citizens Pact to Fight Climate Injustice’, reiterated these themes and added the need to ‘ensure that young people are integrated and represented across all aspects of decision-making’. Also at the New York summit, the Foundation for European Progressive Studies (FEPS) issued a declaration on the theme of ‘United for Climate Justice’. The FEPS document acknowledged that climate change is a ‘triple injustice’ between countries, within countries and across generations (Billingham 2019, 10). It defined climate justice as ‘any social justice issue associated with either the impacts of climate change [or] the preventive measures to address the consequences’ (Ibid). The FEPS project also explicitly linked climate justice with the financial aspect, with a call to ‘[c]limate-proof global investment, finance and trade’, in order to ‘channel finance to activities which support climate mitigation and adaptation, while denying funds to activities which further harm the climate’. These outputs of political parties and party-aligned foundations arguably inform policy-making, as in the context of the informal, three-way coalition of Conservatives, Social Democrats and Liberals in the European Commission and Parliament.

Given the diversity of approaches to climate justice, the question of how to assess the contribution of finance to climate-just outcomes is far from straightforward. As this article is focused on the financial policies and measures of China and the EU, two jurisdictions which are not just parties to the Paris Agreement but which were major protagonists in its negotiation, it is submitted that contribution to the Article 2.1c goal of the Agreement is an appropriate yardstick with which to assess ‘green’ or ‘sustainable’ finance measures. This goal identifies the necessary outcome of financial transition in the context of climate transition, as agreed by parties. The inclusion of this text in the provision on treaty aims had the effect of embedding financial transition in the teleology of the Paris Agreement, a treaty with near-universal membership which addresses both international cooperation and national obligations. Article 2.1c should therefore be seen as the clearest normative statement in international law of the financial transformation required in order to address climate change. Crucially, this goal is universal, unqualified and undifferentiated, such that all parties to the Paris Agreement must work towards it. Just as any financing can contribute to harmful or helpful activities from the perspective of climate mitigation and adaptation, financial frameworks can be assessed according to whether or not they contribute to the redirection of ‘financial flows’ as required by the Paris Agreement.

## The European Union

### Climate justice in the EU

As climate change has become a top policy priority for EU institutions, citizens and many Member States, the concept of climate justice has begun to directly inform legal responses to climate change. While the EU has hosted a rich and diverse debate over climate justice, the dominant approach to climate justice as enacted by EU institutions and Member States, and as confirmed by court judgements, is mostly anthropocentric. It is also holistic, addressing multiple climate impacts on individuals and communities.

Moreover, the just transition concept has arguably become a central feature of EU climate policy. Originally proposed by the union movement (von Lucke, 2019: 23), just transition has more recently been identified as a key consideration for the financial response to climate change. For example, the ‘Investing in a just transition’ project has found that ‘[f]or investors, the just transition provides the framework for connecting climate action with the need for an inclusive economy and sustainable development’ (Robins, Brunsting and Wood 2018, 4). As will be seen, the framing of climate justice has important consequences for the development of climate finance, most obviously through the Just Transition Mechanism but also more broadly.

EU approaches to climate justice embrace both substantive and procedural elements. The substance of climate justice proceeds from recognition that ‘the most vulnerable and poorest in society often have to suffer the greatest impact of the effects of climate change’ (EESC 2017, 3.1). There is therefore a ‘need to ensure the most vulnerable in society do not have to bear an unfair burden and that the cost of a transition to a climate responsive economic model is spread fairly across society’ (Ibid, 4.1.5). Policy responses consistent with climate justice can ‘provide an overarching integrated approach to ensure that the transition to a low carbon economy is achieved in a fair and equitable manner’ (Ibid, 3.3–3.4). As the EESC noted, this must be pursued in relation to areas including consumption, labour, health, energy and migration, where existing inequalities can be affected by climate measures. The European Commission’s proposal for the EGD addressed each of these elements of climate justice, with a major focus on just transition (European Commission 2019).

Responding to the EGD proposal, the European Council drew links between the goal of EU climate neutrality by 2050 and the needs for a just transition and financial support. It ‘recognises the need to put in place an enabling framework that benefits all Member States and encompasses adequate instruments, incentives, support and investments to ensure a cost-effective, just, as well as socially balanced and fair transition, taking into account different national circumstances in terms of starting points’, also acknowledging that the ‘transition will require significant public and private investments’ (European Council 2019, par. 3–4). The European Climate Law, adopted in June 2021, requires the EU to reduce emissions by at least 55% by 2030 compared to 1990 and achieve climate neutrality by 2050 (European Parliament/Council 2021e, Art. 2.1, 4.1). The law requires the Commission to take into account the ‘need to ensure a just and socially fair transition for all’ and ‘fairness and solidarity between and within Member States’ (*inter alia*) in proposing a 2040 EU climate target (Ibid, Art. 4.5), while the public participation clause requires the Commission to ‘engage with all parts of society to enable and empower them to take action towards a just and socially fair transition’ (Ibid, Art. 9.1).

In addition to substantive elements, EU approaches to climate justice include a prominent procedural aspect, embracing participation and consultation, transparency and the availability of litigation (as in the landmark *Urgenda* case, in which the Dutch Supreme Court upheld the finding that the Dutch state was obliged to adhere to a more ambitious mitigation target than that accepted by government: Bouwer 2020; Paiement 2020). The participation of civil society groups has long been important to the crafting of EU climate policy (Derman 2014, 28ff). Stakeholder participation is being further institutionalised through the European Climate Pact (European Commission 2020) and Member State initiatives such as France’s Citizens’ Convention for the Climate. Moreover, as part of the EGD, the Aarhus Regulation may be revised ‘to improve access to administrative and judicial review at EU level for citizens and NGOs’ (European Commission 2019, 23).

Climate justice has also been an important element of the EU’s international stance on climate change (consistent with ‘harm avoidance justice’). While generally not espousing justice in explicit terms, the EU’s consistent advocacy for ambitious and legally binding outcomes has enabled it to build coalitions with the most climate-vulnerable nations, leading to key breakthroughs in UNFCCC negotiations, such as the 2011 Durban platform for a new climate agreement and the 2015 Paris Agreement. The EU’s credibility in aligning itself with climate-vulnerable nations on questions of ambition has rested on its domestic record. There has been much questioning of the limitations of ‘leadership by example’ in the years following the Copenhagen COP (von Luckef 2019: 18). However, others contend that credible implementation remains an important component of EU claims to leadership in international cooperation. As International Relations scholar Yan Xuetong observed, the EU ‘provides authoritative leadership in dealing with climate change, based on its higher achievements in reducing carbon dioxide emissions in the 2010s than other international actors’ (Yan 2019: 16).

### Financing climate justice in the EU

The EU’s legal framework for financing climate justice outcomes continues to develop. The Clean Energy Package of legislation introduced by the Juncker Commission included elements on just transition. The work on sustainable finance, started under the previous Commission, has been continued by the von der Leyen Commission. The EGD and ‘Fit for 55’ packages promise to address in more comprehensive fashion climate justice issues, including just transition.

The initial work of the Juncker Commission on sustainable finance has been expanded by the von der Leyen Commission, as a result of a major shift in European politics favouring more ambitious climate action, after the 2019 election campaign for the European Parliament coincided with a groundswell of public climate activism.

The EGD addresses climate justice through both funding instruments and financial regulations to redirect the flow of capital. A carbon border adjustment mechanism, proposed by the Commission in July 2021, would put a ‘price on imports of a limited number of high-polluting goods based on their carbon content’ (European Commission 2021b, 7). This would discourage carbon leakage and protect EU businesses and workers from unfair competition from foreign competitors which do not operate under sufficient carbon price obligations.

Regarding just transition, the Commission proposed a Just Transition Mechanism (JTM) to ‘focus on the regions and sectors that are most affected by the transition because they depend on fossil fuels or carbon-intensive processes’, and under which ‘[s]upport will be linked to promoting a transition towards low-carbon and climate-resilient activities’ (European Commission 2019, 16). The JTM consists of three ‘pillars’: a Just Transition Fund (JTF), a just transition scheme under InvestEU and a European Investment Bank public sector loan facility. The JTF, financed by €7.5 billion from the 2021–2027 Multiannual Financial Framework (MFF) and €10 billion from the NextGenerationEU (NGEU) pandemic recovery facility (on which see below, “Climate justice at the COVID crossroads” section), will provide mostly grants, with matching financing from other EU funds and Member State co-financing, and would assist ‘territories with high employment in coal, lignite, oil shale and peat production, as well as territories with greenhouse gas-intensive industries, which will be either discontinued or severely impacted by the transition’ (Ibid, 19). The InvestEU scheme would ‘crowd in’ private and public investment in ‘new economic activities’ in affected regions through the provision of guarantees (Ibid, 20–21). The EIB facility would provide concessional loans for public sector investment in the regions (Ibid, 21–22). The Regulation establishing the InvestEU programme was adopted in March 2021 (European Parliament/Council 2021a), while in July 2021, the Council approved the Parliament’s position on the public sector loan facility regulation, paving the way for its adoption (Council of the EU 2021).

The JTF Regulation, adopted in June 2021, calls for Member States to prepare a ‘territorial just transition plan’ for each region seeking JTF support (European Parliament/Council 2021b, art. 11). The plans would include, *inter alia*, description of the national ‘transition process’ towards climate neutrality, assessment of regional ‘transition challenges’ and a ‘description of the expected contribution of the JTF support to addressing the social, demographic, economic, health and environmental impacts of the transition to a climate-neutral economy of the Union by 2050, including the expected contribution in terms of job creation and preservation’ (Ibid).

Clearly, the JTM is but one expression of the climate justice principles discussed above. It is the compromise version of just transition that emerged from the EU legislative process. The JTM has been critiqued on the grounds of both substance (as a ‘restrictive interpretation’ of just transition (Fleming and Mauger 2021, 174)) and procedure (insufficiently providing for community participation in the development of plans (Mauger 2021, 8)). It is nevertheless a major step in concretely integrating climate justice, however imperfectly, into the EU’s legal order.

In addition to the JTM, the 2018 amendments to the EU Emissions Trading System established a Modernisation Fund, which channels two percent of the money raised from auctioning allowances to ‘support a just transition in carbon-dependent regions’ in the ten Member States with the lowest GDP per capita (European Parliament/Council 2018, art. 10d.2).

Aside from just transition, the EGD proposals on sustainable finance largely build on the work initiated by the Juncker Commission. On the spending side, the ‘Sustainable Europe Investment Plan’ will draw on the EU budget, InvestEU guarantees and EIB lending. In November 2019, the EIB adopted a new energy lending policy including a commitment to creating an ‘Energy Transition Package’ and support for just transition more broadly (EIB 2019, 15–16).

On the regulatory side, in November 2019, the EU adopted both a climate benchmarks regulation and a regulation on sustainability-related disclosures. Benchmarks are indices which are used either to set the value of a financial product or to track the performance of an investment fund, e.g. LIBOR for interest rates or Brent Crude for oil. The climate benchmarks regulation creates two new categories of benchmark in order to harmonise benchmark standards and dispel confusion created by a proliferation of low-carbon indices. An ‘EU Climate Transition Benchmark’ is a benchmark where the underlying assets of which are ‘selected, weighted or excluded in such a manner that the resulting benchmark portfolio is on a decarbonisation trajectory’. More ambitiously, an ‘EU Paris-aligned Benchmark’ has underlying assets which ‘are selected, weighted or excluded in such a manner that the resulting benchmark portfolio’s carbon emissions are aligned with the objectives of the Paris Agreement’, the activities of which ‘do not significantly harm other environmental, social and governance (ESG) objectives’ (European Parliament/Council 2019a, art. 1).

The regulation on sustainability-related disclosures in the financial sector requires, among other disclosures, both financial market participants and financial advisers to ‘publish on their websites information about their policies on the integration of sustainability risks in their’ investment decision-making process or investment or insurance advice (as relevant) (European Parliament/Council 2019b, art. 3). Member States are to monitor compliance (Ibid, art. 14).

In June 2020, the EU adopted the taxonomy regulation, which is central to the EU’s ambition to redirect financial flows in support of climate neutrality. The regulation creates a framework for listing officially sanctioned ‘sustainable’ investments and for combating greenwashing. It lists environmental objectives including, *inter alia*, climate mitigation and adaptation (European Parliament/Council 2020, art. 9). The environmental sustainability of an investment is assessed based on how it contributes to specific environmental objectives and whether it causes significant harm to *any* of the environmental objectives (Ibid, art. 3). Transparency requirements are created for various kinds of financial products and investments (Ibid, art. 5–8). The details (or ‘technical screening criteria’: Ibid, art. 19) for whether any specific economic activity contributes to environmental objectives are determined in delegated acts to be adopted by the Commission (Ibid, art. 23). On this, the Commission is being advised by a Platform on Sustainable Finance comprising key agencies and stakeholder groups (Ibid, art. 20). The first delegated act, with technical screening criteria regarding climate mitigation and adaptation, was adopted in June 2021.

The taxonomy criteria will guide the operations of each of the three pillars of the Just Transition Mechanism. Activities supported by the JTF should respect EU climate standards and ‘do no significant harm’ according to the taxonomy regulation (European Parliament/Council 2021b, recital 6). All ‘finance partners’ under the public sector loan facility (i.e. the EIB and other participating institutions) should use the taxonomy and follow its ‘do no significant harm’ principle (European Parliament/Council 2021c, recital 7). The facility’s contribution to the taxonomy’s environmental objectives will also be assessed (Ibid, art. 17.2). The ‘environmental, climate or social impact’ of support under InvestEU will be assessed according to Commission guidance which ‘should appropriately use’ the taxonomy criteria (European Parliament/Council 2021a, recital 13). This Commission ‘sustainability guidance’ will however go beyond the taxonomy’s environmental and ‘no significant harm’ standards to include an explicitly social dimension: ‘estimating the social impact of projects, including on gender equality, on the social inclusion of certain areas or populations and on the economic development of areas and sectors affected by structural challenges such as the need to decarbonise the economy’ (Ibid, art. 8.6).

Further work on sustainable finance in 2021 has included a new sustainable finance strategy (European Commission 2021c), a proposed regulation establishing a European green bond standard (European Commission 2021d) and a proposed directive on corporate sustainability reporting (Commission 2021a). In the context of disclosure and reporting, it is noteworthy that the EU is pioneering the ‘double materiality’ approach, according to which undertakings are obliged to report both their impacts on sustainability and on how sustainability matters affect their performance (see, e.g., European Commission 2021a, art. 1.3).

In addition, the European Central Bank (ECB) has begun to integrate climate considerations into monetary policy. From the beginning of 2021, the ECB has accepted as collateral sustainability-linked bonds, defined as those targeting taxonomy environmental objectives of the climate or environmental Sustainable Development Goals (ECB 2020). In July 2021, the ECB released a climate change action plan which includes, *inter alia*, climate stress-testing of the Eurosystem balance sheet and aligning its corporate asset purchase programme to climate criteria (ECB 2021).

Finally, the sprawling ‘Fit for 55’ package proposed by the Commission in July 2021 to deliver on the new 2030 target contains proposals that would, if adopted, further develop the social aspects of the EU’s financial response to climate change. These include a €72.2 billion ‘Social Climate Fund’ to ‘alleviate the social and distributional burden from the price impacts of the [proposed] emissions trading for the sectors of buildings and road transport’ (European Commission 2021e, 3). While the ultimate outcomes of the ‘Fit for 55’ proposals cannot be predicted, they do indicate that as climate policy becomes more ambitious and encompasses a broader range of economic activity, the need to address questions of fairness becomes correspondingly greater.

To summarise, the EU is developing an increasingly comprehensive regulatory framework to redirect finance flows towards climate-friendly economic activity (while simultaneously targeting other sustainability objectives). EU policy therefore aligns closely with the Paris Agreement’s Article 2.1c goal to transform finance flows, which is unsurprising given that the EU has been a key proponent of this goal, both during the Paris negotiations and after the Paris Agreement entered into force. It can additionally be seen that within its overarching framework for sustainable finance, specific emphasis has been given to public support for just transition outcomes, notably through the JTM and the Modernisation Fund. This operationalisation of the just transition concept responds to both EU ‘cohesion’ policy and demands for intra-EU (and, in a novel departure, even intra-Member State) climate justice. These policy choices highlight the need to address equity and distributional concerns in the redirection of financial flows.

## China

### *Climate justice in **China*

Attention to climate justice in China has been driven by rising public concern about and official acknowledgement of the harmful consequences of the GHG-intensive economy: primarily pollution and its effects on health, but also hazardous work, environmental degradation and corruption. The political system is addressing these concerns under the broad goal of building an ‘ecological civilisation’ (or ‘Xi Jinping Thought on Ecological Civilization’ since its adoption by the current leader). This concept is less anthropocentric than prevailing Western narratives of climate justice (in theory, at least) and also less focused on ‘just transition’ or intra-country distributive justice.

China’s climate policies are largely determined and constrained by the country’s economic development objectives. Development has been China’s main domestic policy goal since the beginning of ‘reform and opening up’ in the late 1970s. The country has been pursuing its two ‘centenary goals’ of building a ‘moderately prosperous society’ by 2020 (declared achieved by Xi at the July 2021 celebration of the Communist Party’s 100th anniversary) and building a ‘great modern socialist country’ by the PRC’s 100th anniversary in 2049 (Nikkei 2021). Specific economic development objectives are defined in and implemented under successive 5-year plans at national and subnational levels. Within this framework, China for decades followed a material- and energy-intensive ‘traditional development model’ (Yi and Liu 2006). This resulted in China becoming by far the largest GHG emitter in absolute terms, with per capita emissions higher than those of the wealthier EU (BBC 2014).

According to this developmentalist logic, the dominating ‘justice’ consideration has been the improvement of the living standards of the Chinese people. Climate policies and commitments that constrained growth were long rejected on this basis. As a senior National Development and Reform Commission (NDRC) official explained in the mid-2000s: ‘China doesn’t want its emission volume to be higher than the United States, but … [t]he priority is to satisfy our basic demand. The economy must develop. China has 1.3 billion people and we have to live’ (Bjørkum, 2005, 57).

As the pollution consequences of China’s economic model grew heavier, official constructions of development broadened to encompass environmental and climate outcomes. Environmental protection has been included in the career incentives of cadres alongside traditional growth targets (Wu and Cao 2021), while the State Council announced that ‘environmental targets would outweigh economic growth measures starting from 2017’ (Li and Shapiro 2020, 59). Nevertheless, as China’s climate targets become more ambitious, the tension with other developmental imperatives is likely to persist. The topic of energy security is an example. While in the long term expanded domestic renewable energy is expected to improve China’s security of supply (IRENA 2019), in the short term, China has responded to energy security risks by ramping up coal capacity and oil and gas production (Meidan 2020; Tu 2020).

The background to Chinese climate policy is the country’s rapid economic growth in the early 2000s powered by fossil fuels, with coal consumption doubling between 2002 and 2008 (Kwon and Hanlon 2016, 1180). The harmful consequences have fallen disproportionately on working people, as mining accidents and other occupational hazards associated with coal-fired energy production claimed thousands of lives each year (Aden and Sinton 2006, 259). Fossil-fuel energy consumption and vehicle exhaust emissions were major sources of the pollution that regularly enveloped Chinese megacities (He 2015, 104). Chinese officials have long been aware of these problems. In 2005, Vice-minister Pan Yue of the then-Environmental Protection Agency warned that China’s economic ‘miracle will end soon, because the environment can no longer keep pace’ (Hilton, 2013, 8). While the predominant global image of China today is of a rising power, Chinese scholars have labelled Chinese residents ‘the most directly affected victims’ of the pollution that has accompanied its growth (Hu et al 2018, 687). In the years following the Beijing Olympics and the ‘blue sky’ days produced for it, concern over air pollution became ubiquitous (Zhang 2018). Since the 2000s, Chinese officials have accepted the need to target environmental protection as well as growth, at least rhetorically. For example, in 2007 Li Yuanchao, then-party secretary of Jiangsu province and a future PRC vice-president, observed that ‘if a child has too many sweets, he will have rotten teeth. GDP is the same. [Sweets are] a good thing but you can’t go to excess’ (McGregor 2011, 90).

During the later years of Hu Jintao’s presidency and under Xi Jinping, Chinese climate policy went from ‘neglect to necessity’ (Kwon and Hanlon 2016, 1178), with the climate transition increasingly prominent in successive 5-year plans. The shift in China’s economic model to emphasise greener development has been buttressed by innovations in the Communist Party’s official political doctrine that highlight the importance of a clean environment. The key concept is ‘ecological civilisation’, which evokes the prior slogan of ‘spiritual civilisation’, used by Deng Xiaoping in the 1980s to ‘signal that modernisation was not just about getting rich’ (Goron 2018, 41). The concept of ecological civilisation was promoted by environmental official Pan Yue in the 2000s. Pan criticised ‘black’, Western industrial civilisation as unsustainable and ‘based on the conquest of nature’. In contrast, ecological civilisation ‘conforms to the law of nature and to the policies of sustainable social, economic and cultural development’. Pan characterised ecological civilisation as distinctively Chinese, referencing the historical legacies of Confucianism, Taoism and Buddhism (Pan 2006). Scholars have pointed to the tension between this theory and China’s actual history (e.g. Hansen, Li and Svarverud 2018, 198). Pan also grounded ecological civilisation in socialism (Geall and Ely 2018, 1184). Ecological civilization has been inserted into both the Party constitution and the state constitution (Wang 2018, 716). The placing of ‘ecological civilization’ in the state constitution’s preamble alongside ‘material civilization’ (*inter alia*) signals a change in China’s development model (Zhao 2021, 17). The concept, which can be traced back to at least 1986 (Barresi 2017, 14), was adopted by Hu Jintao in 2007.

Ecological civilisation (so the *China Daily* averred) is ‘not a term the Party has coined just to fill a theoretical vacancy in its socialism with Chinese characteristics, but rather a future-oriented guiding principle based on the perception of the extremely high price we have paid for our economic miracle’ (People’s Daily 2007). Scholars have emphasised that achieving ecological civilisation requires balancing ‘balancing industrialisation, people’s well-being, and environmental protection’, while avoiding ‘[e]xtreme and unreasonable actions’ such as the local electricity rationing imposed in 2010 to meet 5-year plan targets (Xiao and Zhao 2017, 1008–1009).

Xi Jinping’s September 2020 announcement of new climate targets was framed in the context of ‘building an ecological civilization’ (Xinhua 2020). Xi committed China to peaking CO2 emissions before 2030 and achieving carbon neutrality before 2060. These targets were included in the outline of the 14th 5-year plan (2021–2025), which also set targets for CO2 and energy intensity reductions by 2025 (Xinhua 2021a). The Party has emphasised the ambition of the ‘30/60 targets’, describing them as a ‘tough battle’ and ‘a major test of the party’s capabilities in governing the country’ (Xinhua 2021b).

China’s climate transition has substantial distributional justice implications, even if their relationship to ‘ecological civilisation’ is ambiguous. China faces similar issues to the EU in terms of geographically concentrated coal sector employment (Spencer, Berghmans and Sartor 2017, 22). More generally, it has been observed that the costs of China’s ‘ecological civilisation’ reforms ‘have fallen disproportionately on the weakest parts of society’ (Wang 2018, 760). The official doctrine contains ‘little focus on redistribution’ (Geall and Ely 2018, 1186–87) and has much more to say on topics such as innovation. Others have argued that ‘ecological civilisation’ is a ‘hollow concept’ with little bearing on China’s actual practice of ‘environmental authoritarianism’ (Li and Shapiro 2020, 197). There are nevertheless some references to poverty and disadvantaged central and western districts in key documents (e.g. CPC Central Committee/State Council 2015; Xi 2019), as well as an emerging practice of ‘ecological poverty alleviation’ (People’s Daily 2020). There is also evidence that ‘ecological civilisation’ has been co-opted by local activists in their struggles against environmental injustice (Hansen and Liu 2018, 322).

### *Financing climate justice in **China*

While the EU’s sustainable finance frameworks have explicitly addressed climate justice concerns, the relationship between climate justice and China’s ‘green finance’ policy is more ambiguous. In part, this is because the dominant climate justice narrative in China concerns the inter-state level. For over thirty years, the Chinese state has consistently emphasised the ‘common but differentiated responsibilities’ (UNFCCC 1992, Art. 3.1) of developed and developing countries, including the responsibility of developed countries to provide financial assistance to developing countries (Bo 2013). This stance had greater bearing on China’s domestic climate finance during the years when external financing played a proportionately larger role (e.g. China was the largest recipient of finance under the Kyoto Protocol’s Clean Development Mechanism). It is less relevant to the current situation in which domestic financing dominates Chinese climate projects, while China has itself become a major outbound financier of mitigation and adaptation projects in third countries. Nevertheless, elements of ‘green finance’ have targeted specific aspects of climate justice, including support for victims of disasters and poverty alleviation. More broadly, given China’s status as both the largest GHG emitter and a major international fossil fuel financer, aligning Chinese finance flows to the Article 2.1c goal can be seen as a prerequisite for climate-just outcomes internationally as well as domestically.

Domestically, the Chinese approach to climate finance has undergone significant change. For many years, China was reluctant to commit to mitigation actions that were perceived as economically costly and disruptive, including to employment (Richerzhagen and Scholz 2008, 314). Over time, however, the conditions for policy changed. The relative prices of renewable energies dropped precipitously as against fossil fuels—a phenomenon led in part by the scaling of solar and wind industries in China. By 2015, China’s annual investment in renewable energy constituted 36 per cent of the global total (Manner and Niedermaier 2018, 87). Public concerns about air quality incentivised officials to shutter the most polluting installations and encourage electric transportation. Renewables also contribute to national energy security (Miles 2018, 376–77). Moreover, as Chinese industries moved up the value chain, state economic planning placed increasing emphasis on innovation (Magnus 2018, 146). Priorities for technological leadership included renewables, electric vehicles and sustainable urbanisation. ‘Green development’ became an important component of China’s economic reform and modernisation (State Council/World Bank 2013). The domestic turn to cleaner energy has a complex relationship with China’s external footprint. There have been concerns that China is using its Belt and Road Initiative to ‘dump’ excess steel and coal capacity in participating countries (Magnus 2018, 182). Internally, however, encouragement of ‘green finance’ became a key enabler of both ‘ecological civilisation’ and broader economic goals.

The Chinese government has estimated its annual financial needs for addressing climate change at around 3.7 trillion yuan (approximately $520 billion) between 2016 and 2030, with a ‘shortfall’ of around 1.3 trillion yuan per year (PRC 2018, 152). Most of the shortfall will have to be filled from domestic sources, requiring stimulation of domestic ‘green finance’. The financial policies applied to achieve climate goals build on the ‘financialisation’ of the Chinese economy, which began in the 1990s (Zhang 2019, 213–14). As elsewhere, financialisation in China involved creation of markets for trading securities. Unlike in many other countries, however, financialisation was not accompanied by privatisation of the largest businesses and banks. Rather, ‘[f]inancialization of the state refers to the process in which the Chinese state transforms its management of the economy from administrative intervention and fiscal allocation to supervising its massive state assets according to shareholder value’ (Wang 2015, 621). Chinese financialisation is essentially state-led (for an overview of this phenomenon, see Walter and Howie (2012)). ‘Green finance’ is no exception.

Green credit was the first major green finance policy instrument (Chen et al 2019, 1918) and remains important. In 2007 the then-State Environmental Protection Agency, PBoC and China Banking Regulatory Commission (CBRC) issued opinions on ‘Implementing Environmental Protection Policies and Rules and Preventing Credit Risks’ (SEPA/PBoC/CBRC 2007). This document explained that ‘increased credit risk caused by the closure of polluting enterprises has seriously affected social stability and economic security’ (par. 1). It requires government environmental protection departments at various levels to furnish financial departments with information on both polluting enterprises and ‘environment-friendly’ enterprises (par. 3). Financial institutions must ‘exercise strict control’ over credit allocation based on this information and on state environmental regulations (par. 2). In 2012, the CBRC issued ‘Green Credit Guidelines’, which regulate ‘banking financial institutions’ in greater detail (CBRC 2012, art. 2). Under the Green Credit Guidelines, banks improved both their environmental and financial performance as they became ‘active with regard to integrating environmental risks into their credit risk assessment procedures’ (Weber 2016, 3–4).

Policy development since the mid-2010s has focused on broadening green finance governance. The rationale was explained in the 2015 IISD/State Council report on ‘Greening China’s Financial System’: while green credit was an appropriate tool for the ‘“catching up” stage’ of China’s development, further progress at technological frontiers and involving novel business models can be beyond the expertise of banks to assess. Therefore, greening the full range of financial products could ‘provide a greater diversity of perspectives from institutional investors, intermediaries and risk investors to evaluate green projects, as well as strengthen risk pricing and expand the financial service supply’ (IISD/State Council 2015, 9). Moreover, as PBoC Research Bureau chief economist Ma Jun explained, the green credit policy mostly entailed ‘restrictive measures’, while positive incentives were missing (PBoC/UNEP 2015, xii).

The process that led to China’s current ‘Green Financial System’ policy can be summarised as follows. In April 2015, the PBoC and the UNEP Inquiry released their final report on ‘Establishing China’s Green Financial System’ (PBoC/UNEP 2015). The report made recommendations on specialised investment institutions, fiscal and financial policy support, financial infrastructure and legal infrastructure (Ibid, 3). In September 2015 the CCP Central Committee and State Council proposed the ‘construction of a green financial system’ in their ‘Overall Plan for the Reform of the Ecological Civilization System’. In August 2016, the State Council released the ‘Guidelines for Establishing a Green Financial System’ (PBoC et al. 2016). The Guidelines adopt most of the PBoC/UNEP recommendations.

The Guidelines define ‘green finance’ as ‘economic activities for the purpose of supporting the improvement of environment, responding to climate change, and conserving and efficiently using resources’ (Ibid, par. 1–2). The Guidelines specify actions in the areas of vigorously developing green credit, promoting the securities markets in supporting green investment, establishing green development funds and mobilising private capital through public–private partnerships (PPP), developing green insurance, improving the markets for the trading of environmental rights and interests and creating more financing instruments, vigorously supporting the local governments in developing green finance, advancing international cooperation in green finance and preventing financial risks and strengthening organisation and implementation.

While the Guidelines as a whole are relevant to climate justice, in the sense that transforming finance flows is a prerequisite to a just climate response, certain of the Guidelines address specific justice claims directly. For example, among the guidance on green insurance, ‘[a] catastrophe insurance system relating to climate change shall be established and improved’ (par. 23). Pilot schemes for environmental catastrophe have been launched in several provinces (IIGF/UNEP Inquiry 2017, 21). Moreover, the section on green credit requires institutions to take ‘environmental and social risks’ into account when evaluating credit quality and allocating capital (par. 10), as well as ‘enterprises’ violation of environmental laws and regulations’ (PBoC et al. 2016, par. 11).

As the above indicates, in China the central bank has played and continues to play a more central role in green finance, compared to the EU. In March 2021, the PBoC nominated green finance as a ‘key task’ for the 14th 5-year plan period (PBoC 2021a). Most of the work outlined by the PBoC parallels the EU/ECB actions discussed above: green finance standards including a revised green bond standard, reporting and disclosure, stress-testing and international cooperation. The Bank also flagged more direct market interventions such as preferential interest rates and ‘green special refinancing’ (Ibid; for more on the PBoC’s ‘three functions, five pillars’ approach to green finance development, see People’s Daily 2021).

It is no coincidence that China is using a similar toolkit of ‘green finance’ mechanisms to that of the EU. Chinese ‘green finance’ policies have built upon collaborative studies between Chinese institutions and international, including European, partners. These included the China’s Green Finance Task Force (GFTF), a collaboration of the PBOC and the UNEP Inquiry, which culminated in 2015 (Elliott and Zhang 2019, 396–97), and the collaboration between State Council researchers, the International Institute for Sustainable Development, the UNEP Inquiry and Norway’s Fridtjof Nansen Institute (Zhang 2019, 216–17). The PBoC/UNEP report itemised various EU and Member State laws and initiatives as positive examples (PBoC/UNEP 2015, 8ff). Elliott and Zhang, who analysed the network of Chinese and foreign actors participating in these collaborations, concluded that the GFTF process ‘facilitated transnational policy diffusion via collaborative social construction … and learning’ (2019, 397).

Of course, China cannot be expected to copy EU models of sustainable finance. Rather, ‘Chinese policy-makers regard the EU as a source of guidance, but they adamantly reject any political and legal strictures from European countries’ (Liu, Wu and Wan 2019, 252). Moreover, the particular characteristics of China’s political economy, not least its closed capital account (Zhang 2019, 219), limit convergence. Green finance in China has been a tool for channelling state-owned capital towards environmentally sustainable economic activities. The major growth in green bonds was attributed to issuance by large state-owned banks, fast-track administrative approval of ‘green’ issues and ability to use proceeds to pay down other debt or as working capital (contrary to international standards). The 2016 Guidelines attempt to address this ‘financial statism’ (Zhang 2019, 218–19), nominating mobilisation of private capital as the ‘major purpose of building a Green Financial System’ (PBoC et al. 2016, par. 3).

China has also taken a different path regarding regulation of green bonds (Elliott and Zhang 2019, 399ff). The EIB and China’s Green Finance Committee have set the joint goal of a ‘common language for green finance’, including environmental use of proceeds of green bonds, but so far without result (EIB/GFC 2017). The removal of ‘clean coal’ from the list of projects eligible for green bonds is however an important step towards international standards (Shepherd and Weinland 2020). This was confirmed in the 2021 green bond catalogue jointly issued by the PBoC, NDRC and China Securities Regulatory Commission and in effect from July 2021 (PBoC 2021b). Potentially just as significant is the PBoC’s ‘Assessment Plan for Green Finance of Banking Financial Institutions’, also released in 2021. This document, which expands the performance evaluation of financial institutions from green credit to also encompass green bonds, can be seen as a measure to reduce reliance on green credit and further stimulate green bond growth (PBoC 2021c).

Overall, a 2019 study of green financial development since 2011 concluded that ‘the degree of green financial development in China is relatively low at present’ (He et al. 2019, 979). China’s government has estimated that the green credit provided by the 21 ‘major financial institutions in banking’ in China reached around 7.51 trillion yuan by the end of 2016 or around 8.83 per cent of all loans (PRC 2018, 157). There is also evidence that green finance has developed unevenly in China, with a gap between the more prosperous coastal cities and provinces and inland regions (Liu et al. 2020). The process of aligning finance flows within China with the Article 2.1c goal remains at an early stage, and the absence of a taxonomy-like tool of general application (as opposed to a green bond catalogue) may impede whole-of-economy transformation of finance flows. Evidently, however, the 30/60 targets are providing new impetus, and further initiatives can be expected.

## Comparing the EU and Chinese financial frameworks

As indicated above, the financial policies of the EU and China enact differing conceptions and priorities of climate justice, in the contexts of very different political and economic systems. It is therefore to be expected that there are major differences in the respective frameworks of the two jurisdictions.

As already noted, the Chinese policies do not have a dedicated focus on just transition. This reflects the fact that the just transition concept has not gained traction in the internal Chinese policy debate. As a unitary state, Chinese policy is not subject to the process of negotiation that characterises the EU, which encompasses 27 Member States with very different per capita incomes and energy mixes. In the context of the intra-EU negotiation, the Just Transition Mechanism was a key ‘ask’ of poorer and fossil fuel-reliant Member States that enabled the EU to collectively adopt the ambitions of the European Green Deal. However, just transition is arguably at least as relevant to China as to the EU, given China’s far greater reliance on fossil fuels and coal in particular. This makes just transition a logical topic for future exchanges and potential collaboration between China and the EU.

More broadly, it is noteworthy that the redistributive elements of the EU policies (far from limited to the JTM) mostly do not find parallels in China’s ‘ecological civilization’ concept or in its practical implementation through green finance. At least as far as climate policy is concerned, there is evidently more focus on redistribution in the EU’s ‘social market economy’ (Treaty on European Union, Art. 3.3) than in ‘socialism with Chinese characteristics for a new era’.

Turning to the financial instruments utilised in the EU and China, we see different starting points but an increasing reliance on many common mechanisms. The EU has developed a broad range of financial tools, e.g. pioneering both emissions trading and green bonds. China, with its bank-dominated financial system (Walter and Howie 2012, 27ff), has long been more reliant on green credit. However, since the introduction of the ‘Green Financial System’ framework, China has been expanding its toolkit to include instruments such as carbon trading and green bonds. Both jurisdictions are deepening the procedural justice element transparency through reporting and disclosure requirements. Both the ECB and PBoC are planning to further integrate climate considerations into their macroprudential regulation. China and the EU are also working to identify common green finance standards, with the Chinese side expecting these to be finalised by the end of 2021 (Wang and Luo 2021). While it is unrealistic to expect full convergence between such different systems, these developments indicate the potential for China and the EU to both further bilateral cooperation and contribute to the development of transnational standards aligned to the Article 2.1c goal.

## Climate justice at the COVID crossroads

The COVID-19 pandemic is the greatest global economic shock of recent decades. Mass unemployment and the failure of businesses have required governments around the world to respond with stimulus packages and longer-term planning for economic recovery. This economic shock has placed the global climate response at a crossroads. The International Energy Agency has warned that ‘[g]overnments have a once-in-a-lifetime opportunity to shape a better energy future’ (IEA 2020). How could such an opportunity be realised? Prior to the announcement of most 2020 recovery packages, Hepburn et al. applied the lessons of financial crisis fiscal responses to the current crisis. They concluded that ‘green stimulus policies often have advantages over traditional fiscal stimulus’ (Hepburn et al. 2020, S366). Based on a survey of economic officials and other economists from 53 countries, the authors concluded that the most desirable recovery policies would simultaneously deliver long-run economic multipliers and strong positive climate impacts. They also noted that such recovery policies should also address ‘existing societal and political concerns—such as poverty alleviation, inequality, and social inclusion’ (Ibid, S364). Based on this analysis, Hepburn et al. recommended that recovery packages include clean infrastructure investment, building efficiency retrofits, education and training to address unemployment (including as a result of decarbonisation), natural capital and clean R&D investment and, for lower-to-middle-income countries, rural support spending (Ibid, S374-75).

The EU and China have taken diverging paths. While the EU is doubling down on climate action, China appears to have fallen back on carbon-intensive economic activity. While the pandemic was still ongoing at the time of writing, it was clear that the decisions already taken in response to it will have major ramifications for the pursuit of climate justice in both the EU and China.

Climate action and specific climate justice measures feature prominently in the EU’s pandemic response. In July 2020, the European Council simultaneously agreed to the EU’s 2021–2027 budget (MFF) and its €750 billion pandemic response instrument, NGEU. The Council targeted a recovery that repaired the damage caused by COVID-19 ‘whilst supporting the Union’s green and digital priorities’ (European Council 2020, 2). As discussed above, the Just Transition Mechanism draws funding from both the budget and NGEU. However, the ‘mainstreaming’ of climate across both the MFF and NGEU is far broader. The Council set a ‘climate target’ of 30% of MFF and NGEU expenditure (Ibid, 7).

The main component of NGEU is the €672.5 billion Recovery and Resilience Facility (RRF). To access this facility, each Member State must submit to the Commission a recovery and resilience plan (European Parliament/Council 2021d, Art. 18.1). At least 37% of the plan’s total allocation must be dedicated to climate action, which the Commission is to assess based on a detailed methodology set out in Annex VI of the RRF regulation (Ibid, Art. 19.3(e)). Plans must also observe the ‘no significant harm’ principle of the taxonomy regulation and be consistent with territorial just transition plans (Ibid, Art. 17.3, 18.4(e)).

At the time of writing, the Commission had endorsed the plans of sixteen Member States, in each case necessarily finding that the climate component meets the 37% threshold. According to a Bruegel dataset, the overall allocations in submitted plans correspond well to the recovery policies recommended by Hepburn et al.: 11.41% for clean technologies and renewables, 9.92% for energy efficiency of buildings, 17.83% for sustainable transport, 11.38% for education and training to support digital skills and 7.05% for ‘other green’ investments (Darvas et al. 2021). A separate study of ten Member State plans found that 98% of ‘climate-relevant spending’ (€240 billion) would reduce emissions (Vivid Economics 2021, 4). Many of the proposed reforms and investments tackle specific climate justice elements. For example, both Austria and Greece address energy poverty (Ibid), while Cyprus’ plan explicitly identifies ‘[t]ackling energy poverty in households with disabled people’ as a just transition measure (Republic of Cyprus 2021, 107, 139).

In contrast to the EU, China’s pandemic recovery plans have not included a significant climate focus and have contained few dedicated climate justice measures. Chinese stimulus spending has been conservatively estimated at $731 billion (Vivid Economics/F4B 2021b, 38). Much of the funding has gone to the carbon-intensive industrial sector, while car subsidies have generally not been conditioned on environmental performance (Ibid, 38–39). More positively, stimulus plans have included focus on investment in ‘new infrastructure’, such as 5G (Xu 2020). However, in June 2020, it was reported that over 40GW of new coal capacity had been approved in China in the year-to-date—a greater amount than was approved in 2018 and 2019 combined (Hale and Hook 2020).

The same report carried a statement from central government ministries on the need to ‘consolidate the work of resolving excess coal production’, suggesting central concern at the reliance on coal in China’s COVID-19 economic recovery. Influential officials have called for more ‘green stimulus’ (e.g. GFLP 2020) but without apparent success. Instead, the steel and cement industries reported higher growth in 2020 than in 2019, reflecting a fossil fuel-intensive response to pandemic disruption (Du, Jia and Wei 2021). The challenge is particularly acute in China because its ‘green finance’ framework is focused heavily on debt (credit and bonds), for which demand has ‘significantly decreased’ as part of a general credit market contraction (UNEP Inquiry 2020, 8). There have been some examples of ‘green’ stimulus, including the launch of a $12 billion Green Development Fund. Investment in ‘building renovation for older people’, including energy efficiency improvements (Vivid Economics/F4B 2021b, 40), could be considered a climate justice measure.

The February 2021 ‘Greenness of Stimulus Index’ released by Vivid Economics and the Finance for Biodiversity Initiative confirmed the divergence between the EU and China. The study found the EU to have the ‘greenest’ pandemic stimulus measures of any G20 participant (Vivid Economics/F4B 2021a, 4). In contrast, China’s stimulus plans were rated as having a net negative impact on the environment, with relaxation of environmental reporting requirements and ‘unconditional’ stimulus for polluting industries. The study nevertheless noted that China’s score had improved in recent months due to ‘stricter emission reduction targets and ambitions for massive renewable energy deployment’ (Ibid, 15). A July 2021 report from the same organisations found that, overall, ‘greenness of stimulus is improving slightly over time’, with the trend observable in China due to the launch of its carbon market, new emission targets and announced phase-out of fossil-fuel vehicles (Vivid Economics/F4B 2021b, 4, 11).

This research indicates that while the EU’s response thus far to the pandemic’s economic impact is accelerating efforts to align finance flows with climate goals, China’s response has been ambivalent at best and has largely buttressed prior models of carbon-intensive activity. The EU’s recovery plans have also directly addressed aspects of climate justice, which are mainstreamed beyond the dedicated JTM, while a Chinese focus on these matters is not apparent. In essence, the EU has prioritised a green and digital recovery, while China has prioritised a fast recovery, which entailed a rapid reopening of industry and protection of employment. However, while the Chinese stimulus can therefore be seen as a missed opportunity, it is likely to be eclipsed by new initiatives to meet the 30/60 targets.

## Conclusion

Climate justice has been a concept with a protean nature, shifting form according to the objectives of those who invoke it. Climate justice has unquestionably risen up the political agenda in recent years, and the essential *injustice* of unchecked climate change unites many different perspectives and interests. As for the details of how to address this injustice and on what basis, diversity reigns. China and the EU approach the domestic enactment of climate justice with perspectives that are as different as their political systems and histories would suggest. In the EU, just transition is a key enabler of climate neutrality, and well-established procedural norms of participation, social dialogue and transparency are indispensable. In China, climate justice is subsumed within ‘ecological civilisation’, perhaps the ‘most significant Chinese state-initiated imaginary of our global future’ (Hansen, Li and Svarverud 2018, 196).

However climate justice is defined, it is clear that a transformation of finance flows will be required to achieve it. In the domain of ‘sustainable’ or ‘green’ finance, China and the EU have been drawing from—and further developing—a common toolkit of systemic interventions to redirect the financial system, prominently including standardisation, disclosure requirements and carbon markets. Illustrating the versatility of these financial mechanisms, they are being implemented in starkly different economic and political systems. Adapting the Chinese saying ‘same bed, different dreams’ (同床异梦), it is a case of ‘common mechanisms, different perspectives’.

The pandemic has placed aspirations for climate justice at a crossroads. Massive fiscal intervention by governments will set the direction of economies—and emissions—for years to come. So far, EU and Chinese reactions to the pandemic look different in climate terms. The EU is doubling down on climate action, while the Chinese response is ambiguous. EU pandemic stimulus has been quite well aligned with the Paris Agreement’s transformative Article 2.1c goal—the achievement of which is a prerequisite for meeting climate justice demands in the long term. In China, the picture is more mixed but may still develop in a positive direction, as the country prepares to achieve the 30/60 targets. Greater engagement on sustainable finance between the EU and China could assist in generating momentum for green recovery, including by exploring opportunities for coordination and alignment. This engagement could be pursued bilaterally as well as in fora such as the International Platform on Sustainable Finance and the Coalition of Finance Ministers for Climate Action.
